# Supplementary material for: Complex water networks visualized by cryogenic electron microscopy of RNA
Source: Nature. 2025 Mar 11;642(8066):250–9. doi: 10.1038/s41586-025-08855-w (PMC12137144; doi:10.1038/s41586-025-08855-w)
Supplement: Supplementary file 1 — Supplementary Tables 1–3 and full descriptions for Supplementary Videos 1 and 2 and Supplementary Data. [file 41586_2025_8855_MOESM1_ESM.pdf]

nature portfolio

https://doi.org/10.1038/s41586-025-08855-w

Supplementary information

Complex water networks visualized by cryogenic electron microscopy of RNA

In the format provided by the authors and unedited

Supplemental Information for:

Complex water networks visualized by cryogenic electron microscopy of RNA

Rachael C. Kretsch[1#], Shanshan Li[2#], Grigore Pintilie[3#], Michael Z. Palo[4], David A. Case[5], Rhiju Das[1,6,7*], Kaiming Zhang[2*], Wah Chiu[1,3,8,9*]

[1]Biophysics Program, Stanford University School of Medicine, Stanford, California, USA.
[2]Department of Urology, The First Affiliated Hospital of USTC, MOE Key Laboratory for Cellular Dynamics, Center for Advanced Interdisciplinary Science and Biomedicine of IHM, Division of Life Sciences and Medicine, University of Science and Technology of China, Hefei 230001, China.
[3]Department of Bioengineering and James Clark Center, Stanford University School of Medicine, Stanford, California, USA.
[4]Department of Structural Biology, Stanford University School of Medicine, Stanford, California, USA.
[5]Department of Chemistry & Chemical Biology, Rutgers University, Piscataway, New Jersey, USA.
[6]Department of Biochemistry, Stanford University School of Medicine, Stanford, California, USA.
[7]Howard Hughes Medical Institute, Stanford University, Stanford, California, USA.
[8]Department of Microbiology and Immunology, Stanford University School of Medicine, Stanford, California, USA.
[9]Division of CryoEM and Bioimaging, SSRL, SLAC National Accelerator Laboratory, Menlo Park, California, USA.
[#] These authors contributed equally to this work.
* Corresponding authors: rhiju@stanford.edu, kmzhang@ustc.edu.cn, and wahc@stanford.edu

Page 3: **Supplemental Table 1:** Relative hydration of various domains of the ribozyme.

Page 4: **Supplemental Table 2:** Agreement with the immediate solvent shell of the 2.2 Å cryo-EM map where the RNA atoms are well resolved and almost identical between the two independent maps

Page 5-8: **Supplemental Table 3.** Atoms identified by nucleotide analog interference mapping to have strong interference of catalytic activity, as labeled by references[47–52], and the interaction observed in the structures presented.

Supplemental Video 1. Animation of *Tetrahymena* ribozyme cryo-EM map at 2.2 Å resolution, with modeled RNA, ions, and water molecules. Zoom-in sequences illustrate panels C, H, E, and F from **Fig. 3**.

Supplemental Video 2. Animation of *Tetrahymena* ribozyme cryo-EM maps at 2.2 and 2.3 Å resolution, with modeled RNA, ions, and water molecules. Maps are displayed at 3σ above background. Illustrates the 3-dimensional context for **Fig. 5B-E**.

Source Data File 1: Summary of water and Mg^{2+} ions modeled by SWIM.
For labelling overlap the following symbols are used
# - atom found that is superimposable and binds same RNA atoms
* - atom found that is superimposable
^ - atom found that binds same RNA atoms

Source Data File 2: Water binding sites in molecular dynamics simulation, their occupancy in molecular dynamics simulation, and whether they are found in the SWIM/cryo-EM models.

Source Data File 3: Mg^{2+} ion binding sites in molecular dynamics simulation, their occupancy in molecular dynamics simulation, and whether they are found in the SWIM/cryo-EM models. Sites with > 30% occupancy have been annotated with whether ions were placed at that position initially.

Supplemental Table 1: Relative hydration of various domains of the ribozyme.

Region	Nucleotides considered *	Number of nucleotides	Average Q-score	Average number of consensus water bound (± s.e.m)	Accuracy † of predicted TIP4P-D water density (± s.e.m)
P2 & P2.1	27, 31-62, 76, 90-95	40	0.76	0.7 ± 0.2	0.37 ± 0.03
P3	96-106, 272-278	18	0.81	4.4 ± 0.4	0.43 ± 0.02
P4 & P5	107-117, 119-121, 123-124, 200-205, 209-214, 259-260	30	0.74	1.3 ± 0.4	0.33 ± 0.03
P5abc	125-129, 132-149, 151, 154-178, 180-196	66	0.76	1.0 ± 0.3	0.41 ± 0.03
P5b-P6 TTR	150, 152, 153, 223, 224, 248, 250	7	0.77	1.1 ± 0.9	0.58 ± 0.05
Catalytic site	206-208, 261, 262, 304-308	10	0.75	2.8 ± 0.9	0.34 ± 0.04
P6	215-222, 225-228, 245-247, 251-258	23	0.77	3.3 ± 0.7	0.45 ± 0.04
P7	263-271, 309-314	15	0.79	2.7 ± 0.6	0.39 ± 0.03
P8	279-286, 294-303	18	0.76	1.7 ± 0.6	0.42 ± 0.07
P9	315-331, 404-406	21	0.74	0.05 ± 0.1	0.37 ± 0.07
P9.1 & P9.2	332, 334-348, 353-366, 401-402	32	0.70	0.2 ± 0.1	0.23 ± 0.03
All resolved		280	0.75	1.4 ± 0.2	0.38 ± 0.02

s.e.m, standard error of the mean

* Nucleotides with Q-score > 0.6 in both maps are considered.

† Accuracy, calculated per nucleotide, is the normalized area under the precision recall curve, comparing the MD probability to density >3σ in the 2.2 Å map which is 1.8-3.5 Å aways from the nucleotide (AU-PRC^{MD}, Online Methods).

Supplemental Table 2: Agreement with the immediate solvent shell of the 2.2 Å cryo-EM map where the RNA atoms are well resolved and almost identical between the two independent maps

	Cross-correlation	Mutual information	Area under precision-recall curve	Mathews correlation coefficient
Maximal value	1.00	0.5020	1.00	1.00
Random *	0.00	0.0003	0.13	0.03
Independent map †	0.90	0.2059	0.75	0.63
SWIM model	0.25	0.0019	0.31	0.34
Molecular dynamics	0.23	0.0131	0.31	0.26

* 2.2 Å cryo-EM map shuffled, represents the lower bound of agreement

† 2.3 Å cryo-EM, represents the experimental uncertainty

Supplemental Table 3. Atoms identified by nucleotide analog interference mapping to have strong interference of catalytic activity, as labeled by references47–52, and the interaction observed in the structures presented.

∗ When only one atom is listed, the literature identified this atom as interfering with catalysis when substituted, however, did not identify what atom it was interacting with.

† The following interaction were not included in the table as they are not applicable in apo state studied here:

- J4/5 - P1 helix Szewczak et al 1998, Strobel et al 1998, Strauss-Soukup and Strobel 2000
- J8/7 - P1 helix Szewczak et al 1998, Strauss-Soukup and Strobel 2000
- G22-G27 Strobel and Shetty 1997, Ortoleva-Donnelly et al Biochemistry 1998
- 300-302 interactions Szewczak et al 1998, Ortoleva-Donnelly et al RNA 1998

Interacting atoms ∗,†	Reported in	Interaction in structure
A256 N6	Ortoleva-Donnelly et al RNA 1998	**Water-mediated interaction**<br>U300 OP
A308 N7	Ortoleva-Donnelly et al RNA 1998	**Water-mediated interaction**<br>A306 N3
A308 N6	Ortoleva-Donnelly et al RNA 1998	Direct H-bond<br>U267 O4<br>**Water-mediated interaction**<br>A306 N3
A114 O2’	Ortoleva-Donnelly et al RNA 1998	**No interaction**<br>Possible water-mediated interaction with A207 N1, weak density.
A114 N7	Ortoleva-Donnelly et al RNA 1998	**No interaction**<br>In plane with A106 (H-bond of A114 N6 and A206 N3) could have water/ion mediated interaction, but not clear density
A207 N7	Ortoleva-Donnelly et al RNA 1998	**No interaction**<br>In-plane with A113 (H-bond of A113 N6 and A207 N3) could have water/ion mediated interaction, but not clear density.

**Interacting atoms ∗,†**	**Reported in**	**Interaction in structure**
A256 N7	Ortoleva-Donnelly et al RNA 1998	**No interaction**<br>Density supports a water bound to this atom which would coordinate the Mg2+ ion which coordinates the OP of A256, U273.
A261 N7	Ortoleva-Donnelly et al RNA 1998	**No interaction**<br>Density supports ion-mediated interaction with A265 OP, modeled as Mg2+ ion, could be a monovalent ion.
A306 N7	Ortoleva-Donnelly et al RNA 1998	**No interaction**<br>Density supports water/ion mediated interaction with A261 OP.
A308 N7	Ortoleva-Donnelly et al RNA 1998	**Ion-mediated interaction**<br>U307 OP
A95 HN6	Ortoleva-Donnelly et al RNA 1998	Direct H-bond<br>U56 O2’
A95 N7	Ortoleva-Donnelly et al RNA 1998	Direct H-bond<br>U56 HO2’
A97 O2’	Ortoleva-Donnelly et al RNA 1998	Direct H-bondU59 O2’G92 N2
G111 N2	Strobel and Shetty 1997, Ortoleva-Donnelly et al Biochemistry 1998	Direct H-bond<br>C208 O2
G112 N2	Strobel and Shetty 1997, Ortoleva-Donnelly et al Biochemistry 1998	Direct H-bond<br>C209 O2
A210 N6	Strobel et al 1998, Ortoleva-Donnelly et al RNA 1998	Direct H-bond<br>A46 N3
A210 O2’	Ortoleva-Donnelly et al RNA 1998	Direct H-bond<br>C211 O2’
G212 N2	Szewczak et al 1998	Direct H-bond<br>C109 O2<br>A184 N3
A218 N6	Ortoleva-Donnelly et al RNA 1998	Direct H-bond<br>U273 O2’
A218 O2’	Ortoleva-Donnelly et al RNA 1998	Direct H-bond<br>C102 O2’

Interacting atoms ∗,†		Reported in	Interaction in structure
A219 N6		Ortoleva-Donnelly et al RNA 1998	Direct H-bond G254 O2’ and N3
A256 HO2’		Ortoleva-Donnelly et al RNA 1998	Direct H-bond G272 O2’
A261 O2’		Szewczak et al 1998, Ortoleva-Donnelly et al RNA 1998	Direct H-bond G264 OP
A270 N7		Ortoleva-Donnelly et al RNA 1998	Direct H-bond A103 N6
A270 O2’		Ortoleva-Donnelly et al RNA 1998	Direct H-bond G272 N7
G303 N2		Strobel and Shetty 1997, Ortoleva-Donnelly et al Biochemistry 1998, Szewczak et al 1998	Direct H-bond A302 OP G303 OP
A306 O2’		Ortoleva-Donnelly et al RNA 1998	Direct H-bond A261 N3
A97 N7	U300 H3	Szewczak et al 1998, Ortoleva-Donnelly et al RNA 1998	Direct H-bond
A97 HN6	U300 O4	Szewczak et al 1998, Ortoleva-Donnelly et al RNA 1998	Direct H-bond
A114 HN6	A206 N3	Strobel et al 1998, Ortoleva-Donnelly et al RNA 1998	Direct H-bond
A114 HN6	A206 O2’	Strobel et al 1998, Ortoleva-Donnelly et al RNA 1998	Direct H-bond
G150 HO2’	A152 N7	Strauss-Soukup and Strobel 2000	Direct H-bond
G150 HN2	A153 OP	Strauss-Soukup and Strobel 2000	Direct H-bond
G150 HN2	A153 N7	Strauss-Soukup and Strobel 2000	Direct H-bond
A151 N1	A248 HN6	Strauss-Soukup and Strobel 2000	Direct H-bond
A151 HN6	A248 N1	Strauss-Soukup and Strobel 2000	Direct H-bond
A152 HO2’	U224 HO2’	Strauss-Soukup and Strobel 2000	Direct H-bond
A152 N3	U224 HO2’	Strauss-Soukup and Strobel 2000	Direct H-bond
A152 HN6	G250 O2’	Strauss-Soukup and Strobel 2000	Direct H-bond
A153 O2’	C223 HO2’	Strauss-Soukup and Strobel 2000	Direct H-bond
A153 HO2’	C223 O2	Strauss-Soukup and Strobel 2000	Direct H-bond
A153 N1	G250 HO2’	Strauss-Soukup and Strobel 2000	Direct H-bond

**Interacting atoms ∗,†**		**Reported in**	**Interaction in structure**
A153 N3	G250 HN2	Strauss-Soukup and Strobel 2000	Direct H-bond
A207 HN6	A213 N3	Strobel et al 1998, Ortoleva-Donnelly et al RNA 1998	Direct H-bond
A207 HN6	A213 O2’	Strobel et al 1998, Ortoleva-Donnelly et al RNA 1998	Direct H-bond
C209 HN4	U305 O4	Szewczak et al 1998	Direct H-bond
C223 O2	G250 HN2	Strauss-Soukup and Strobel 2000	Direct H-bond
C223 N3	G250 N1	Strauss-Soukup and Strobel 2000	Direct H-bond
C223 HN4	G250 O6	Strauss-Soukup and Strobel 2000	Direct H-bond
U224 N3	A238 N7	Strauss-Soukup and Strobel 2000	Direct H-bond
U224 O2	A248 HN6	Strauss-Soukup and Strobel 2000	Direct H-bond
