# Supplementary material for: Complex water networks visualized by cryogenic electron microscopy of RNA
Source: Nature. 2025 Mar 11;642(8066):250–9. doi: 10.1038/s41586-025-08855-w (PMC12137144; doi:10.1038/s41586-025-08855-w)
Supplement: Supplementary file 2 — Reporting Summary [file 41586_2025_8855_MOESM2_ESM.pdf]

nature portfolio

Corresponding author(s): 2024-06-11682
Rhiju Das, Kaiming Zhang, Wah Chiu

Last updated by author(s): Feb 3, 2025

Reporting Summary

Nature Portfolio wishes to improve the reproducibility of the work that we publish. This form provides structure for consistency and transparency in reporting. For further information on Nature Portfolio policies, see our Editorial Policies and the Editorial Policy Checklist.

Statistics

For all statistical analyses, confirm that the following items are present in the figure legend, table legend, main text, or Methods section.

n/a	Confirmed	
☒	☐	The exact sample size (*n*) for each experimental group/condition, given as a discrete number and unit of measurement
☒	☐	A statement on whether measurements were taken from distinct samples or whether the same sample was measured repeatedly
☐	☒	The statistical test(s) used AND whether they are one- or two-sided<br>*Only common tests should be described solely by name; describe more complex techniques in the Methods section.*
☒	☐	A description of all covariates tested
☒	☐	A description of any assumptions or corrections, such as tests of normality and adjustment for multiple comparisons
☐	☒	A full description of the statistical parameters including central tendency (e.g. means) or other basic estimates (e.g. regression coefficient) AND variation (e.g. standard deviation) or associated estimates of uncertainty (e.g. confidence intervals)
☐	☒	For null hypothesis testing, the test statistic (e.g. *F*, *t*, *r*) with confidence intervals, effect sizes, degrees of freedom and *P* value noted<br>*Give P values as exact values whenever suitable.*
☒	☐	For Bayesian analysis, information on the choice of priors and Markov chain Monte Carlo settings
☒	☐	For hierarchical and complex designs, identification of the appropriate level for tests and full reporting of outcomes
☐	☒	Estimates of effect sizes (e.g. Cohen's *d*, Pearson's *r*), indicating how they were calculated

Our web collection on statistics for biologists contains articles on many of the points above.

Software and code

Policy information about availability of computer code

Data collection	EPU software (Thermo Fisher Scientific, version 2.7)
Data analysis	MotionCor2 (1.2.6), CTFFIND4 (4.1.12), EMAN2 (20200925), Relion (3.0), cryoSPARC (3.2.0), phenix (1.14; including the molprobity plugin), ISOLDE (ChimeraX v1.6.1), Amber (20), MDAnalysis (2.7), Q-score https://github.com/gregdp/mapq (1.9.12), Segger https://github.com/gregdp/segger (v2.9.7), manuscript scripts: https://github.com/DasLab/Water-CryoEM-ribozyme

For manuscripts utilizing custom algorithms or software that are central to the research but not yet described in published literature, software must be made available to editors and reviewers. We strongly encourage code deposition in a community repository (e.g. GitHub). See the Nature Portfolio guidelines for submitting code & software for further information.

Data

Policy information about availability of data

All manuscripts must include a data availability statement. This statement should provide the following information, where applicable:

- Accession codes, unique identifiers, or web links for publicly available datasets
- A description of any restrictions on data availability
- For clinical datasets or third party data, please ensure that the statement adheres to our policy

Cryo-EM maps have been deposited in the wwPDB OneDep System under EMD accession codes 42499, 42498 for the 2.2 and 2.3 Å map respectively. The atomic models associated with the 2.2 Å map are deposited in the PDB under accession codes 9CBU for the models with only the consensus waters and ions and 9CBX for

the model with all automatically identified waters and ions. The atomic models associated with the 2.2 Å map are deposited in the PDB under accession codes 9CBW for the models with only the consensus waters and ions and 9CBY for the model with all automatically identified waters and ions. The cryo-EM raw movies and particle stacks have been deposited to the Electron Microscopy Public Image Archive (EMPIAR) under accession code 11844. Simulations can be found at Stanford Digital Repository https://doi.org/10.25740/sw275qs6749.

Research involving human participants, their data, or biological material

Policy information about studies with human participants or human data. See also policy information about sex, gender (identity/presentation), and sexual orientation and race, ethnicity and racism.

Reporting on sex and gender	N/A
Reporting on race, ethnicity, or other socially relevant groupings	N/A
Population characteristics	N/A
Recruitment	N/A
Ethics oversight	N/A

Note that full information on the approval of the study protocol must also be provided in the manuscript.

Field-specific reporting

Please select the one below that is the best fit for your research. If you are not sure, read the appropriate sections before making your selection.

☒ Life sciences ☐ Behavioural & social sciences ☐ Ecological, evolutionary & environmental sciences

For a reference copy of the document with all sections, see nature.com/documents/nr-reporting-summary-flat.pdf

Life sciences study design

All studies must disclose on these points even when the disclosure is negative.

Sample size	Sample sizes were not predetermined. The number of particles used for cryo-EM reconstruction was limited by the 48 hours of collection time, the particles were sufficient for the desired resolution. The number of magnesium ions and water ions analyzed were limited by resolution of the map, there was insufficient magnesiums for a full analysis, hence the full analysis was only conducted on water.
Data exclusions	In cryo-EM, particles were excluding during 2D classification and 3D reconstruction as is standard in the field. For molecular dynamics frames were excluded that deviated too far from the starting orientations, as defined by RMSD.
Replication	In cryo-EM the standard is not to do full replicates for reconstruction, but instead reconstruct two halves of the data independently, this was done and the cryo-EM maps were "replicable" up to 2.2 and 2.3 Å. For the molecular dynamics, 30 replicates were conducted in total, and these are reproducible for the properties analysed here-in.
Randomization	This is irrelevant to the study as their were no groupings. For the molecular dynamics, initial water and metal placements were randomized for some simulations.
Blinding	Blinding was not relevant for this study, structure of this RNA was known to all researcher involved in solving this structure.

Reporting for specific materials, systems and methods

We require information from authors about some types of materials, experimental systems and methods used in many studies. Here, indicate whether each material, system or method listed is relevant to your study. If you are not sure if a list item applies to your research, read the appropriate section before selecting a response.

Materials & experimental systems

n/a	Involved in the study
☒	☐ Antibodies
☒	☐ Eukaryotic cell lines
☒	☐ Palaeontology and archaeology
☒	☐ Animals and other organisms
☒	☐ Clinical data
☒	☐ Dual use research of concern
☒	☐ Plants

Methods

n/a	Involved in the study
☒	☐ ChIP-seq
☒	☐ Flow cytometry
☒	☐ MRI-based neuroimaging

Plants

Seed stocks	N/A
Novel plant genotypes	N/A
Authentication	N/A
